# Maternal and Birth Characteristics and Childhood Embryonal Solid Tumors: A Population-Based Report from Brazil

**DOI:** 10.1371/journal.pone.0164398

**Published:** 2016-10-21

**Authors:** Neimar de Paula Silva, Rejane de Souza Reis, Rafael Garcia Cunha, Júlio Fernando Pinto Oliveira, Marceli de Oliveira Santos, Maria S. Pombo-de-Oliveira, Beatriz de Camargo

**Affiliations:** 1 Pediatric Hematology and Oncology Program, Research Center, Instituto Nacional de Câncer, Rio de Janeiro-RJ, Brazil; 2 Divisão de Vigilância e Análise de Situação Coordenação de Prevenção e Vigilância, Instituto Nacional do Câncer, Rio de Janeiro-RJ, Brazil; Ospedale Pediatrico Bambino Gesu, ITALY

## Abstract

**Background:**

Several maternal and birth characteristics have been reported to be associated with an increased risk of many childhood cancers. Our goal was to evaluate the risk of childhood embryonal solid tumors in relation to pre- and perinatal characteristics.

**Methods:**

A case-cohort study was performed using two population-based datasets, which were linked through R software. Tumors were classified as central nervous system (CNS) or non-CNS-embryonal (retinoblastoma, neuroblastoma, renal tumors, germ cell tumors, hepatoblastoma and soft tissue sarcoma). Children aged <6 years were selected. Adjustments were made for potential confounders. Odds ratios (OR) with 95% confidence intervals (CI) were computed by unconditional logistic regression analysis using SPSS.

**Results:**

Males, high maternal education level, and birth anomalies were independent risk factors. Among children diagnosed older than 24 months of age, cesarean section (CS) was a significant risk factor. Five-minute Apgar ≤8 was an independent risk factor for renal tumors. A decreasing risk with increasing birth order was observed for all tumor types except for retinoblastoma. Among children with neuroblastoma, the risk decreased with increasing birth order (OR = 0.82 (95% CI 0.67–1.01)). Children delivered by CS had a marginally significantly increased OR for all tumors except retinoblastoma. High maternal education level showed a significant increase in the odds for all tumors together, CNS tumors, and neuroblastoma.

**Conclusion:**

This evidence suggests that male gender, high maternal education level, and birth anomalies are risk factors for childhood tumors irrespective of the age at diagnosis. Cesarean section, birth order, and 5-minute Apgar score were risk factors for some tumor subtypes.

## Introduction

Childhood embryonal solid tumors occur more frequently at younger ages suggesting that antenatal, perinatal, and early postnatal exposures may play a part in its pathogenesis [1, 2]. Represents a heterogeneous group of cancer composed of undifferentiated cells that resemble tissues from the developing embryo and fetus suggesting that defects in tissue growth pathways and their differentiation during prenatal/postnatal period would promote tumor genesis [3]. The principal types of embryonal tumors are neuroblastoma, nephroblastoma, retinoblastoma, hepatoblastoma and significant cases of childhood central nervous system tumors [4]. Incidence rates vary worldwide and in Brazil it was observed regional variations according to socioeconomic status. Data showed a significant correlation between socioeconomic status and incidence rate of neuroblastoma and retinoblastoma [5].

Most childhood cancers occur sporadically and etiological evidence for causes is poor. Hereditary or familial factors are evident in 10% of cases [6]. Several maternal and birth characteristics are reported to be associated with an increased risk of many childhood cancers. Birth order, maternal age, mode of delivery, Apgar score, and congenital anomalies are described as risk factors for some solid tumors [2, 7–12]. Associations between congenital anomalies and embryonal tumors are well established [2, 12]. Risk associations were found between birth weight and several childhood cancers, and described with greater certainty in leukemia and renal tumors [13]. Birth characteristics probably represent interactions between genetic susceptibility and perinatal environmental causes [2]. Several case-control studies have been published, but the rarity of childhood cancers makes it difficult to identify potential etiologic clues. We conducted a population-based study in Brazil to investigate the association between maternal, perinatal, and birth characteristics and childhood embryonal solid tumors.

## Materials and Methods

### Study Design and Population

A case-cohort study was performed by selecting cases and controls within the same total population at baseline which allows the advantages of both cohort and case-control designs [14]. Data were obtained from the Live Birth Information System (SINASC) from 14 cities with Population-Based Cancer Registries (PBCR). The quality of the Brazilian population-based registries has improved and is considered good [15–19]. Cases among children with solid tumors born after 1999 and diagnosed between 2000 and 2010 were selected (n = 566). Both datasets were linked through probabilistic data linkage using RStudio software [20, 21]; and 395 (70%) of the initially identified were successfully matched to their birth records. Full details are described elsewhere [22]. The peak incidence rate of embryonal tumors is among children 1 to 4 years old therefore to analyze risk factors, we further selected children aged <6 years who were diagnosed with embryonal tumors. We classified tumors as central nervous system (CNS) (n = 119) or non-CNS embryonal (retinoblastoma, n = 28; neuroblastoma, n = 64; renal tumors, n = 62; germ cell tumors, n = 32; hepatoblastoma, n = 6; or soft tissue sarcoma, n = 29) tumors. Four controls per case were chosen by systematic random sampling (n = 1580) from the SINASC data source, and were ordered by birth year and gender for all solid tumors including children aged >5 years [18]. For these analyses, the same control group (n = 1580) and 340 cases were selected. Variables available from SINASC database were gender, 5-minute Apgar score, mode of delivery, congenital anomalies, birth order, birth weight, maternal age at child's birth, and maternal education.

### Statistical Analysis

Odds ratios (OR) and 95 percent confidence intervals (CIs) were computed by unconditional logistic regression analysis using SPSS version 21.0. The analysis of both all tumors and for each specific tumor group always included the total control group for comparison. Apgar score was categorized into three levels (0–5, 6–8, 9–10) and in two levels (≤8 versus >8) [23]. To test the sensitivity of the results we ran analyzes for 12, 24 and 36 months of age and results were significant for 24 months of age (data not shown). Separate analysis were conducted for age at diagnosis (≤24 months or >24 months) and for tumor subtype (CNS or non-CNS embryonal tumors). All variables were included in a logistic regression model and adjustments were made using only variables with p values ≤0.20.

All data were kept strictly confidential, ensuring anonymity.

The study was approved by the Research Ethical Committee of Instituto Nacional de Cancer (INCA) ref: 13596513.7.0000.5274.

## Results

Maternal and perinatal characteristics of the controls and cases are presented in Table 1. The presence of birth anomalies was detected in 7 cases and 6 controls. Birth anomalies were classified as described in the International Classification of Diseases tenth revision (ICD-10). Among the 7 cases birth anomalies were described as unspecified congenital anomaly of the foot (n = 1), Down syndrome (n = 1), macrocephaly (n = 1), unspecified syndrome (n = 1), spina bifida (n = 1), unspecified brain abnormality (n = 1), and congenital anomaly of the male genital tract (n = 1).

**Table 1:** Sociodemographic, maternal, pre and perinatal characteristics of childhood embryonal tumors and controls, Brazil, 2000–2010.

	Controls	Cases	CNS tumors	Non-CNS embryonal tumors*
	n(%)	n(%)	n(%)	n(%)
Gender				
Female	774 (49.0)	138 (40.6)	43 (36.1)	95 (43.0)
Male	805 (51.0)	202 (59.4)	76 (63.9)	126 (57.0)
Missing	1 (0.0)	-	-	-
Race				
Non-White	734 (46.4)	171 (50.3)	64 (53.8)	107 (48.4)
White	742 (47.0)	155 (45.6)	51 (42.8)	104 (47.1)
Missing	104 (6.6)	14 (4.1)	4 (3.4)	10 (4.5)
Geographic region				
North	204 (12.9)	43 (12.6)	17 (14.3)	26 (11.8)
Northeast	512 (32.4)	110 (32.4)	32 (26.9)	78 (35.3)
Southeast	436 (27.6)	96 (28.2)	34 (28.6)	62 (28.1)
South	348 (22.0)	75 (22.1)	29 (24.4)	46 (20.8)
Midwest	80 (5.1)	16 (4.7)	7 (5.9)	9 (4.1)
Maternal age (years)				
<25	830 (52.5)	168 (49.4)	63 (52.9)	105 (47.5)
25–35	636 (40.3)	149 (43.8)	46 (38.7)	103 (46.6)
>35	108 (6.8)	23 (6.8)	10 (8.4)	13 (5.9)
Missing	6 (0.4)	-	-	-
Maternal education (years)				
<3	241 (15.2)	34 (10.0)	10 (8.4)	24 (10.9)
04–11	1075 (68.0)	227 (66.8)	77 (64.7)	150 (67.9)
≥12	221 (14.0)	69 (20.3)	28 (23.5)	41 (18.5)
Missing	43 (2.7)	10 (2.9)	4 (3.4)	6 (2.7)
Birth order				
First	541 (34.2)	125 (36.8)	40 (33.6)	85 (38.5)
Two or higher	909 (57.5)	191 (56.2)	68 (57.1)	123 (55.7)
Missing	130 (8.3)	24 (7.0)	11 (9.2)	13 (5.9)
Mode of delivery				
Vaginal	931 (58.9)	169 (49.7)	55 (46.2)	114 (51.6)
Cesarean	646 (40.9)	171 (50.3)	64 (53.8)	107 (48.4)
Missing	3 (0.2)	-	-	-
Birth anomalies				
no	1503 (95.1)	323 (95.0)	114 (95.8)	209 (94.6)
yes	6 (0.4)	7 (2.1)	1 (0.8)	6 (2.7)
Missing	71 (4.5)	10 (2.9)	4 (3.4)	6 (2.7)
Duration of gestation (weeks)				
<37	91 (5.8)	12 (3.5)	7 (5.9)	5 (2.3)
37–41	1454 (92.0)	323 (95.0)	110 (92.4)	213 (96.4)
>41	20 (1.3)	2 (0.6)	1 (0.8)	1 (0.5)
Missing	15 (0.9)	3 (0.9)	1 (0.8)	2 (0.9)
5-minute Apgar				
0–5	18 (1.1)	1 (0.3)	1 (0.8)	-
6–8	142 (9.0)	38 (11.2)	14 (11.8)	24 (10.9)
9–10	1313 (83.1)	289 (85.0)	98 (82.4)	191 (86.4)
Missing	107 (6.8)	12 (3.5)	6 (5.0)	6 (2.7)
Birth weight (g)				
< 2500	119 (7.5)	14 (4.1)	6 (5.0)	8 (3.6)
2500–4000	1390 (88.0)	307 (90.3)	105 (88.2)	202 (91.4)
> 4000	71 (4.5)	19 (5.6)	8 (6.7)	11 (5.0)
Birth weight by gestational age				
SGA	273 (17.3)	55 (16.2)	19 (16.0)	36 (16.3)
AGA	1213 (76.8)	263 (77.3)	90 (75.6)	173 (78.3)
LGA	78 (4.9)	19 (5.6)	9 (7.6)	10 (4.5)
Missing	16 (1.0)	3 (0.9)	1 (0.8)	2 (0.9)

AGA, appropriate for gestational-age; LGA, large for gestational-age; SGA, small for gestational age;

*Include retinoblastoma, neuroblastoma, renal tumors, germ cell tumors, hepatoblastoma and soft tissue sarcoma.

**Fig 1:**
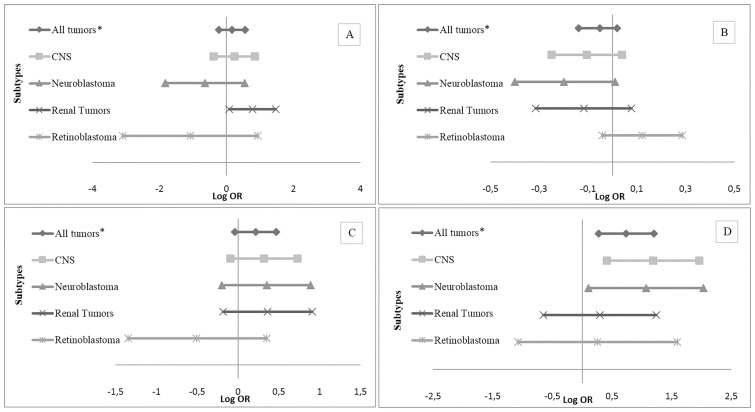
Adjusted Risk Estimates for Maternal and Perinatal Characteristics and Pediatric Tumors According to Subtypes, Brazil 2000–2010. (A) Adjusted^a^ risk estimates for Apgar 5-level ≤8 and pediatric tumors. (B) Adjusted^b^ risk estimates for continuous birth order-per order of 1 and pediatric tumors. (C) Adjusted^b^ risk estimates for mode of delivery-cesarean and pediatric tumors. (D) Adjusted^b^ risk estimates for maternal education level ≥12 years and pediatric tumors. OR—Odds Ratio; CI—Confidence Interval; ^a^Adjusted by sex, birth weight and birth anomalies; ^b^ Adjusted by maternal education, sex, birth weight and birth anomalies; *Include CNS tumors, retinoblastoma, neuroblastoma, renal tumors, germ cell tumors, hepatoblastoma and soft tissue sarcoma.

Table 2 shows the crude and adjusted ORs and 95% CIs between all tumors and sociodemographic and maternal pre- and perinatal variables. Male sex, high maternal education level, and birth anomalies were independent risk factors. Continuous maternal age (per 5 years increased) shown a modest association with all embryonal tumors together, despite not significant. As shown in Fig 1A, an Apgar score ≤8 was an independent risk factor for renal tumors (OR = 2.17 (95% CI 1.08–4.35)). A decreasing risk with increasing birth order was observed for all tumor types except retinoblastoma. Among children with CNS tumors, neuroblastoma and renal tumors there were a decreased risk with increasing birth order (OR = 0.90 (95% CI 0.78–1.04)); (OR = 0.82 (95% CI 0.67–1.01)); (OR = 0.89 (95% CI 0.73–1.08)), respectively (Fig 1B). Delivery by cesarean section showed a marginally significantly increased OR for all tumors except retinoblastoma. Among children with CNS tumors, neuroblastoma and renal tumors there were an increased risk with delivery by cesarean section (OR = 1.37 (95% CI 0.91–2.07)); (OR = 1.42 (95% CI 0.82–2.43)); (OR = 1.44 (95% CI 0.83–2.48)), respectively **(**Fig 1C). Maternal education level higher or equal to 12 years showed a significant increase in the odds for all tumors together, as well as for the group of CNS tumors (OR = 3.28 (95% CI 1.51–7.13)) and for neuroblastoma (OR = 2.91 (95% CI 1.11–7.61)) (Fig 1D).

**Table 2:** Risk estimates for sociodemographic, maternal, pre and perinatal factors and embryonal tumors, Brazil 2000–2010.

	All Tumors			
	%controls	%cases	Crude OR (95% CI)	Adjusted OR (95% CI)
Race^a^				
Non-White	49.7	52.5	1.11 (0.87–1.41)	0.98 (0.75–1.26)
White	50.3	47.5	1.00	1.00
Maternal age (years)^a^				
per 5 years	-	-	1.07 (0.98–1.17)	1.06 (0.97–1.16)
<25	52.7	49.4	0.86 (0.67–1.10)	0.89 (0.69–1.15)
25–35	40.4	43.8	1.00	1.00
>35	6.9	6.8	0.90 (0.56–1.47)	1.00 (0.61–1.66)
Maternal education (years)^b^				
<3	15.7	10.3	1.00	1.00
04–11	69.9	68.8	1.49 (1.01–2.20)	1.47 (0.99–2.18)
≥12	14.4	20.9	2.21 (1.41–3.46)	2.09 (1.32–3.32)
Birth order^a^				
per order of 1	-	-	0.94 (0.76–1.16)	0.95 (0.87–1.02)
First	37.3	39.6	1.09 (0.85–1.41)	0.96 (0.73–1.25)
Two or higher	62.7	60.4	1.00	1.00
Mode of delivery^a^				
Vaginal	59	49.7	1.00	1.00
Cesarean	51	50.3	1.45 (1.15–1.84)	1.24 (0.96–1.60)
Birth anomalies^c^				
no	99.6	97.9	1.00	1.00
yes	0.4	2.1	5.42 (1.81–16.20)	5.24 (1.72–15.9)
Duration of gestation (weeks)^a^				
<37	5.8	3.6	0.59 (0.32–1.09)	0.65 (0.31–1.36)
37–41	92.9	95.8	1.00	1.00
>41	1.3	0.6	0.45 (0.10–1.93)	0.41 (0.08–1.93)
5-minute Apgar^a^				
0–5	1.2	0.3	0.25 (0.03–1.89)	0.35 (0.04–2.69)
6–8	9.6	11.6	1.21 (0.83–1.77)	1.27 (0.84–1.90)
9–10	89.1	88.1	1.00	1.00

OR—Odds Ratio; CI—Confidence Interval;

^a^ Adjusted by maternal education, sex, birth weight and birth anomalies;

^b^ Adjusted by sex, birth weight and birth anomalies;

^c^ Adjusted by maternal education, sex and birth weight.

When we stratified according to age at diagnosis (≤24 months versus >24 months) higher maternal education level (≥12 years) and birth anomalies continued to be an independent risk factors among children aged ≤24 months.

Among children diagnosed older than 24 months of age, delivery by cesarean section was a significant risk factor (Table 3). Five-minute Apgar score was analyzed according to age at diagnosis (<6 months or ≥6 months) and a score ≤8 had a 2-fold risk in children aged <6 months, despite not significant (Table 4).

**Table 3:** Risk estimates for sociodemographic, maternal, pre and perinatal factors and embryonal tumors according to age strata, Brazil 2000–2010.

	All Tumors					
	Diagnosed ≤24 months old			Diagnosed > 24 months old		
	cases	Crude OR (95% IC)	Adjusted OR (95% IC)	cases	Crude OR (95% IC)	Adjusted OR (95% IC)
Race^a^	174			152		
Non-White		1.18 (0.86–1.62)	1.00 (0.71–1.41)		1.03 (0.74–1.44)	0.98 (0.69–1.39)
White		1.00	1.00		1.00	1.00
Maternal age (years)^a^	181			159		
per 5 years increase		1.06 (0.94–1.19)	1.06 (0.94–1.19)		1.08 (0.95–1.22)	1.08 (0.95–1.23)
<25		0.84 (0.61–1.16)	0.89 (0.63–1.25)		0.88 (0.63–1.24)	0.88 (0.62–1.25)
25–35		1.00	1.00		1.00	1.00
>35		0.95 (0.51–1.77)	1.00 (0.52–1.91)		0.85 (0.42–1.70)	0.90 (0.45–1.83)
Maternal education (years)^b^	175			155		
<3		1.00	1.00		1.00	1.00
04–11		1.39 (0.83–2.33)	1.29 (0.76–2.18)		1.61 (0.93–2.76)	1.58 (0.92–2.73)
≥12		2.72 (1.53–4.85)	2.48 (1.38–4.46)		1.63 (0.84–3.15)	1.62 (0.84–3.14)
Birth order^a^	166			150		
per order of 1		0.95 (0.85–1.05)	0.96 (0.86–1.06)		0.92 (0.82–1.04)	0.93 (0.83–1.04)
First		1.00 (0.71–1.39)	0.82 (0.57–1.17)		1.21 (0.86–1.71)	1.13 (0.79–1.61)
Two or higher		1.00	1.00		1.00	1.00
Mode of delivery^a^	181			159		
Vaginal		1.00	1.00		1.00	1.00
Cesarean		1.39 (1.02–1.89)	1.09 (0.78–1.54)		1.53 (1.10–2.12)	1.47 (1.04–2.07)
Birth anomalies^c^	174			156		
no		1.00	1.00		1.00	1.00
yes		10.4 (3.48–31.60)	10.6 (3.40–33.10)		-	-
5-minute Apgar^a^	174			154		
0–5		0.47 (0.06–3.59)	0.68 (0.08–5.34)		-	-
6–8		1.20 (0.73–1.98)	1.26 (0.74–2.15)		1.22 (0.72–2.06)	1.28 (0.74–2.21)
9–10		1.00	1.00		1.00	1.00

OR—Odds Ratio; CI—Confidence Interval;

^a^ Adjusted by maternal education, sex, birth weight and birth anomalies;

^b^ Adjusted by sex, birth weight and birth anomalies;

^c^ Adjusted by maternal education, sex and birth weight.

**Table 4:** Risk estimates for embryonal tumors according to 5-minute Apgar score, by age at diagnosis, Brazil 2000–2010.

Age at diagnosis	5-min. Apgar	Crude OR (95% IC)	Adjusted^a^ OR (95% IC)
< 6 months			
	0–8	2.05 (0.93–4.53)	1.99 (0.81–4.89)
	9–10	1.0	1.0
≥ 6 months			
	0–8	0.99 (0.66–1.49)	1.10 (0.72–1.68)
	9–10	1.0	1.0

OR—Odds Ratio; CI—Confidence Interval;

^a^Adjusted by maternal education, sex, birth weight and birth anomalies.

## Discussion

Childhood embryonal tumors are frequently diagnosed before children reach the age of 5 years indicating that there are factors involved in utero or during early postnatal life. We selected children from two good quality Brazilian population-based datasets [15–19].

The sample size among specific cancer types became small and our results were limited, which occurs with most studies assessing childhood solid tumors. Pediatric cancers comprise a heterogeneous group and it is unclear whether subgroups should be analyzed together. Causal associations with childhood cancer have begun to be documented [2]. We found an increased risk of childhood cancer among cases with congenital anomalies and high maternal education levels.

Maternal education has been used a proxy for socioeconomic status, though it is not perfect. It is a variable with good completeness in the SINASC dataset [24]. In our series, higher education level (≥12 years of education) was 14.0% in the control group versus 20.3% in the case group. Higher education level was an independent risk factor for all tumors together, as well as among CNS tumors and neuroblastoma. Maternal education has been described with 90% of agreement for 12 years of schooling [25]. Studies done by IBGE in Brazil evaluating in self-reported questionnaire regarding demographic issues reports that overestimated is only on the lowest category of maternal education (http://www.ibge.gov.br). We believe that our result is reliable because it was on the highest category. Maternal education is consider a indicator of social background which can be associated with a variety of health-related factors including risk factors associated with childhood cancer as occupational exposures, dietary patterns, exposure to infectious, immunization, breastfeeding [26–28]. And it has been reported as a confounding factor [26, 27]. A recent paper has shown that genome-wide association was associated with the numbers of years of schooling completed [29]. Another item that requires attention is that immunization is correlated with education background and incomplete immunization has already been described associated with embryonal tumors [28]. Human Development Index (HDI) was similar between the different cities that were included on our study so we believe that migration does not interfere on education background [30]. Several studies have shown no impact of residential mobility regarding environment issues [31, 32]. Unfortunately data from SINASC regarding occupational exposures is incomplete and it was not evaluated in our study [24].

One of the strongest risk factors for childhood cancer is being born with a congenital anomaly. Carcinogenesis and congenital anomalies may have a common basis in some pediatric cancers [9, 12, 33–35]. Among our sample, 13 cases/controls had a congenital anomaly noted at birth. The field for recording birth defects at SINASC is composed of an open-ended question and a field for the description of the birth defect is coded according to the ICD-10. We observed a high ICD-10 ‘not otherwise specified’ among our reported cases, not allowing for more information. In an evaluation of data from SINASC in a hospital in the city of Campinas (São Paulo State), a 46.8% under reporting of all birth defects was observed, indicating that the SINASC database needs improvement to collect information on the prevalence of birth defects [36]. This is an important limitation reported by others. Birth anomalies can be diagnosed at birth but minor anomalies are more common diagnosed latter in life [35]. Down syndrome is a well-established risk factor for infant leukemia whereas the association with solid tumors is uncommon [37, 38]. In our series, a case with macrocephaly had a CNS tumor, which may be a reverse causation [39]. Five cases with germ cell tumors (n = 32) was described with birth anomalies as unspecified brain abnormality, spina bifida, congenital anomaly of the male genital tract, unspecified syndrome and Down syndrome. Spina bifida was more common in children with cancer than among population-based controls [33]. Among solid tumors associated with Down syndrome germ cell tumors are the most described [40, 41]. One case with renal tumor had an unspecified congenital anomaly of the foot. Hemihypertrophy is part of several syndromes associated with embryonal tumors [42]. The unspecified congenital anomaly of the foot could be a signal of these syndromes.

The association between cesarean section and childhood cancer is inconsistent [11]. Cesarean section presented a slightly significant risk only among children diagnosed older than 24 months of age. Some studies suggest a higher risk of neuroblastoma and leukemia [8, 43]. We do not have information on elective or emergency cesarean delivery at SINASC, and this may be an important factor involved in the mechanism of the association [43]. Our data should be treated with caution given that Brazil has one of the highest rates of cesarean deliveries, especially in the South and Southeast regions, and these rates may be associated with the availability of perinatal care services [44]. Among children diagnosed older than 24 months of age, cesarean section was associated with an increased risk of around 50% (OR = 1.47 (95% CI 1.04–2.07)). Children with neuroblastoma showed a strong association with cesarean section, despite the association not being significant. Among Brazilian incidence rates, a high incidence of neuroblastoma has been described in the South region. The high incidence of neuroblastoma was correlated with socioeconomic status [5]. The South and Southeast Brazilian regions have the highest HDI among the different regions [30]. Maternal education was used as a proxy of socioeconomic status and was also a high risk factor for neuroblastoma.

Bilateral and unilateral retinoblastoma were analyzed together. Bilateral retinoblastoma varies little worldwide, whereas unilateral retinoblastoma has a higher incidence in many developing countries suggesting environmental exposure contributing to its causation. [45, 46]. Unfortunately, in our PBCR we do not have the information on laterality. The risk of retinoblastoma was protected by cesarean section, despite not significant. The presence of Human Papilloma Virus (HPV) has been described in retinoblastoma tumors [47] and maternal transfer could be a possible route of transmission [48]. Children born by cesarean section could be protected from HPV infection.

Birth order may be a marker of different hormonal exposures to the fetus, and higher birth order children may present with higher levels of microchimerism [49]. Moreover, birth order has been used as a proxy for postnatal infectious exposures [7]. Birth order was calculated from the number of previous pregnancies, counting both living and dead children plus one; however, this may affect the accuracy of birth order data. We observed a slightly protective correlation with increased birth order, which was non-significant in all tumors except for retinoblastoma which could be lack of genetic counseling. The risk of bilateral retinoblastoma has been described as decreasing with increasing birth order [7].

Different biologic pathways may be responsible for the association between Apgar score and childhood cancer. The 5-min Apgar score is a predictor of neonatal mortality and neurologic outcomes. A low Apgar score is a marker of suboptimal fetal environment and may be associated with compromised immune responses against tumors [50, 51]. Among our cases, a low 5-minute Apgar score on overall cancer risk was strongest for cancers diagnosed before 6 months of age as others have described [10]. The 5-minute Apgar score ≤8 was an independent risk factor for renal tumors. The association between 1-minute Apgar score and Wilms’ tumor has been described in some studies [52, 53], and the 5-minute Apgar score has only been associated with Wilms’ tumor in Nordic countries [10, 54].

Birth weight has been documented as a risk factor for several tumor types, including tumors occurring in adults [55]. Our data has been described elsewhere and suggests that increased birth weight was associated with childhood solid tumor development [22], which was used for adjustments.

Chromosome-number abnormalities have been associated with altered recombination and increased maternal age. The most important factor linked to chromosomal aneuploidy in women is advancing maternal age. It is well established that chromosome-number abnormalities in offspring occur more frequently as maternal age advances [56]. In our series, maternal age was a modest risk factor for the development of embryonal tumors, despite not significant.

This study has the advantage of population-based birth and cancer registries from Brazil. No single cancer registry exits countrywide. People who moved from their city of birth to another city either those that were not born in there city which developed cancer would not have been identified in the study. This is one of our greatest limitations as has been described by others [35, 57]. We lost around 30% of cases identified in PBCR. Brazil has a continental dimension and around 35% of population lives out of birth city [58]. This effect which may bias our data is difficult to evaluate, but we believe that the migration occurs equally between cases and controls. The quality of the data in the PBCR database has significantly improved, which can be observed through the International Agency of Research on Cancer assessment [17]. The SINASC database is recognized as having good to excellent completeness, with consistent information [19, 24]. Another limitation is the lack information on risk factors after birth. In conclusion, even if based on a small sample size and different tumor types, some elevated risks seem to be consistent.

### Declaration of Interest

The authors report no conflicts of interest. The authors alone are responsible for the content and writing of the paper.

## References

[1] Autrup H. Transplacental transfer of genotoxins and transplacental carcinogenesis. Environ Health Perspect. 1993 7;101 Suppl 2:33–8. 8243402 10.1289/ehp.93101s233 PMC1519962

[2] Spector LG, Pankratz N, Marcotte EL. Genetic and nongenetic risk factors for childhood cancer. Pediatr Clin North Am. 2015 2;62(1):11–25. 10.1016/j.pcl.2014.09.013 25435109 PMC4384439

[3] Dehner LP. The evolution of the diagnosis and understanding of primitive and embryonic neoplasms in children: living through an epoch. Mod Pathol. 1998 7;11(7):669–85. 9688189

[4] Gatta G, Ferrari A, Stiller CA, Pastore G, Bisogno G, Trama A, et al Embryonal cancers in Europe. Eur J Cancer. 2012 7;48(10):1425–33. 10.1016/j.ejca.2011.12.027 22357215

[5] De Camargo B, de Oliveira Ferreira JM, de Souza Reis R, Ferman S, de Oliveira Santos M, Pombo-de-Oliveira MS. Socioeconomic status and the incidence of non-central nervous system childhood embryonic tumours in Brazil. BMC Cancer. 2011;11:160 10.1186/1471-2407-11-160 21545722 PMC3112157

[6] Saletta F, Dalla Pozza L, Byrne JA. Genetic causes of cancer predisposition in children and adolescents. Transl Pediatr. 2015 4;4(2):67–75. 10.3978/j.issn.2224-4336.2015.04.08 26835363 PMC4729088

[7] Von Behren J, Spector LG, Mueller BA, Carozza SE, Chow EJ, Fox EE, et al Birth order and risk of childhood cancer: a pooled analysis from five US States. Int J Cancer. 2011 6 1;128(11):2709–16. 10.1002/ijc.25593 20715170 PMC3008504

[8] Urayama KY, Von Behren J, Reynolds P. Birth characteristics and risk of neuroblastoma in young children. Am J Epidemiol. 2007 3 1;165(5):486–95. 10.1093/aje/kwk041 17164463

[9] Fisher PG, Reynolds P, Von Behren J, Carmichael SL, Rasmussen SA, Shaw GM. Cancer in children with nonchromosomal birth defects. J Pediatr. 2012 6;160(6):978–83. 10.1016/j.jpeds.2011.12.006 22244463 PMC4490790

[10] Li J, Cnattingus S, Gissler M, Vestergaard M, Obel C, Ahrensberg J, et al The 5-minute Apgar score as a predictor of childhood cancer: a population-based cohort study in five million children. BMJ Open. 2012;2(4). 10.1136/bmjopen-2012-001095 PMC3425910 22874628

[11] Momen NC, Olsen J, Gissler M, Cnattingius S, Li J. Delivery by caesarean section and childhood cancer: a nationwide follow-up study in three countries. Bjog. 2014 10;121(11):1343–50. 10.1111/1471-0528.12667 24521532

[12] Dawson S, Charles AK, Bower C, de Klerk NH, Milne E. Risk of cancer among children with birth defects: a novel approach. Birth Defects Res A Clin Mol Teratol. 2015 4;103(4):284–91. 10.1002/bdra.23364 25808250

[13] O'Neill KA, Murphy MF, Bunch KJ, Puumala SE, Carozza SE, Chow EJ, et al Infant birthweight and risk of childhood cancer: international population-based case control studies of 40 000 cases. Int J Epidemiol. 2015 2;44(1):153–68. 10.1093/ije/dyu265 25626438

[14] Szklo M, Nieto FJ. Epidemiology Beyond the Basics. Gaithersburg, Maryland: Aspen; 2000 495 p.

[15] Ferreira JS, Vilela MB, Aragao PS, Oliveira RA, Tine RF. [Evaluation of the quality of information: linkage between SIM and SINASC in Jaboatao dos Guararapes, Pernambuco State]. Cien Saude Colet. 2011;16 Suppl 1:1241–6. 21503472 10.1590/s1413-81232011000700056

[16] Guerra FA, Llerena JC Jr., Gama SG, Cunha CB, Theme Filha MM. [Birth defects in Rio de Janeiro, Brazil: an evaluation through birth certificates (2000–2004)]. Cad Saude Publica. 2008 1;24(1):140–9. 18209842 10.1590/s0102-311x2008000100014

[17] Forman D, Bray F, Brewster D, Gombe-Mbalawa C, Kohler B, Piñeros M, et al Cancer Incidence in Five Continents, Vol. X Lyon: IARC; 2013 Available: http://ci5.iarc.fr. 10.1002/ijc.29670 26135522

[18] The Cancer Atlas. Geórgia, Atlanta: American Cancer Society; 2014 Available: http://canceratlas.cancer.org/.

[19] Frias PG, Szwarcwald CL, Lira PI. [Evaluation of information systems on live births and mortality in Brazil in the 2000s]. Cad Saude Publica. 2014 10;30(10):2068–280. 25388310 10.1590/0102-311x00196113

[20] R Core Team. R: A Language and Environment for Statistical Computing Vienna, Austria: R Foundation for Statistical Computing; 2014 Available: http://www.R-project.org/.

[21] Borg A, Sariyar M. RecordLinkage: Record Linkage in R 2015. Available: http://CRAN.R-project.org/package=RecordLinkage.

[22] De Paula Silva N, Reis RS, Cunha RG, Oliveira JFP, Lima FCS, Pombo-de-Oliveira S, et al Birth Weight and Risk of Childhood Solid Tumors in Brazil: a Record Linkage Between Population-Based Datasets. Pan American Journal Of Public Healthy. 2016; 40(x):xxx–xxx. 10.26633/RPSP.2017.14 PMC6660849 28444001

[23] Apgar V. A proposal for a new method of evaluation of the newborn infant. Curr Res Anesth Analg. 1953 Jul-Aug;32(4):260–7. 13083014

[24] Romero DE, Cunha CB. [Evaluation of quality of epidemiological and demographic variables in the Live Births Information System, 2002]. Cad Saude Publica. 2007 3;23(3):701–14. 17334583 10.1590/s0102-311x2007000300028

[25] Querec LJ. Comparability of reporting between the birth certificate and the National Natality Survey. Vital Health Stat 2 1980 (83):1–44. 7395112

[26] Carozza SE, Puumala SE, Chow EJ, Fox EE, Horel S, Johnson KJ, et al Parental educational attainment as an indicator of socioeconomic status and risk of childhood cancers. Br J Cancer. 2010 6 29;103(1):136–42. 10.1038/sj.bjc.6605732 20531410 PMC2905284

[27] Onubogu CU, Onyeka IN, Esangbedo DO, Ndiokwelu C, Okolo SN, Ngwu EK, et al Changes in breastfeeding and nutritional status of Nigerian children between 1990 and 2008, and variations by region, area of residence and maternal education and occupation. Paediatr Int Child Health. 2016 1 29:1–12. 10.1179/2046905515Y.0000000048 26212771

[28] Sankaran H, Danysh HE, Scheurer ME, Okcu MF, Skapek SX, Hawkins DS, et al The Role of Childhood Infections and Immunizations on Childhood Rhabdomyosarcoma: A Report From the Children's Oncology Group. Pediatr Blood Cancer. 2016 9;63(9):1557–62. 10.1002/pbc.26065 27198935 PMC4955701

[29] Okbay A, Beauchamp JP, Fontana MA, Lee JJ, Pers TH, Rietveld CA, et al Genome-wide association study identifies 74 loci associated with educational attainment. Nature. 2016 5 26;533(7604):539–42. 10.1038/nature17671 27225129 PMC4883595

[30] Atlas of Human Development, Brazil. 2013. Available: http://www.atlasbrasil.org.br/2013/. Accessed July, 30, 2016.

[31] Lupo PJ, Symanski E, Chan W, Mitchell LE, Waller DK, Canfield MA, et al Differences in exposure assignment between conception and delivery: the impact of maternal mobility. Paediatr Perinat Epidemiol. 2010 3;24(2):200–8. 10.1111/j.1365-3016.2010.01096.x 20415777

[32] Chen L, Bell EM, Caton AR, Druschel CM, Lin S. Residential mobility during pregnancy and the potential for ambient air pollution exposure misclassification. Environ Res. 2010 2;110(2):162–8. 10.1016/j.envres.2009.11.001 19963212

[33] Narod SA, Hawkins MM, Robertson CM, Stiller CA. Congenital anomalies and childhood cancer in Great Britain. Am J Hum Genet. 1997 3;60(3):474–85. 9042906 PMC1712528

[34] Partap S, MacLean J, Von Behren J, Reynolds P, Fisher PG. Birth anomalies and obstetric history as risks for childhood tumors of the central nervous system. Pediatrics. 2011 9;128(3):e652–7. 10.1542/peds.2010-3637 21824884 PMC3164097

[35] Botto LD, Flood T, Little J, Fluchel MN, Krikov S, Feldkamp ML, et al Cancer risk in children and adolescents with birth defects: a population-based cohort study. PLOS ONE. 2013;8(7):e69077 10.1371/journal.pone.0069077 23874873 PMC3714243

[36] Luquetti DV, Koifman RJ. Quality of reporting on birth defects in birth certificates: case study from a Brazilian reference hospital. Cad Saude Publica. 2009 8;25(8):1721–31. 19649413 10.1590/s0102-311x2009000800008

[37] Hitzler JK, Zipursky A. Origins of leukaemia in children with Down syndrome. Nat Rev Cancer. 2005 1;5(1):11–20. 10.1038/nrc1525 15630411

[38] Hasle H, Clemmensen IH, Mikkelsen M. Risks of leukaemia and solid tumours in individuals with Down's syndrome. Lancet. 2000 1 15;355(9199):165–9. 10.1016/S0140-6736(99)05264-2 10675114

[39] Wilne S, Collier J, Kennedy C, Koller K, Grundy R, Walker D. Presentation of childhood CNS tumours: a systematic review and meta-analysis. Lancet Oncol. 8 England2007 p. 685–95. 10.1016/S1470-2045(07)70207-3 17644483

[40] Hasle H. Pattern of malignant disorders in individuals with Down's syndrome. Lancet Oncol. 2001 7;2(7):429–36. 10.1016/S1470-2045(00)00435-6 11905737

[41] Kobayashi T, Sakemi Y, Yamashita H. Increased incidence of retroperitoneal teratomas and decreased incidence of sacrococcygeal teratomas in infants with Down syndrome. Pediatr Blood Cancer. 2014 2;61(2):363–5. 10.1002/pbc.24693 23904199

[42] Clericuzio CL. Recognition and management of childhood cancer syndromes: a systems approach. Am J Med Genet. 1999 6 25;89(2):81–90. 10559762 10.1002/(sici)1096-8628(19990625)89:2<81::aid-ajmg5>3.0.co;2-i

[43] Francis SS, Selvin S, Metayer C, Wallace AD, Crouse V, Moore TB, et al Mode of delivery and risk of childhood leukemia. Cancer Epidemiol Biomarkers Prev. 2014 5;23(5):876–81. 10.1158/1055-9965.EPI-13-1098 24618997

[44] Gomes UA, Silva AA, Bettiol H, Barbieri MA. Risk factors for the increasing caesarean section rate in Southeast Brazil: a comparison of two birth cohorts, 1978–1979 and 1994. Int J Epidemiol. 1999 8;28(4):687–94. 10480697 10.1093/ije/28.4.687

[45] Agboola AO, Adekanmbi FA, Musa AA, Sotimehin AS, Deji-Agboola AM, Shonubi AM, et al Pattern of childhood malignant tumours in a teaching hospital in south-western Nigeria. Med J Aust. 2009 1 5;190(1):12–4. 19120001 10.5694/j.1326-5377.2009.tb02254.x

[46] Leal-Leal C, Flores-Rojo M, Medina-Sanson A, Cerecedo-Diaz F, Sanchez-Felix S, Gonzalez-Ramella O, et al A multicentre report from the Mexican Retinoblastoma Group. Br J Ophthalmol. 2004 8;88(8):1074–7. 10.1136/bjo.2003.035642 15258028 PMC1772266

[47] Orjuela M, Castaneda VP, Ridaura C, Lecona E, Leal C, Abramson DH, et al Presence of human papilloma virus in tumor tissue from children with retinoblastoma: an alternative mechanism for tumor development. Clin Cancer Res. 2000 10;6(10):4010–6. 11051250

[48] Bhuvaneswari A, Pallavi VR, Jayshree RS, Kumar RV. Maternal transmission of human papillomavirus in retinoblastoma: A possible route of transfer. Indian J Med Paediatr Oncol. 2012 10;33(4):210–5. 10.4103/0971-5851.107080 23580821 PMC3618642

[49] Adams KM, Nelson JL. Microchimerism: an investigative frontier in autoimmunity and transplantation. Jama. 2004 3 3;291(9):1127–31. 10.1001/jama.291.9.1127 14996783

[50] Gilstrap LC 3rd, Hauth JC, Hankins GD, Beck AW. Second-stage fetal heart rate abnormalities and type of neonatal acidemia. Obstet Gynecol. 1987 8;70(2):191–5. 3601281

[51] Ekbom A. The developmental environment and the early origins of cancer In: Gluckman P, Hanson M. eds. Developmental origins of health and disease. Cambridge: Cambridge University Press, 2006:415–25.

[52] Puumala SE, Soler JT, Johnson KJ, Spector LG. Birth characteristics and Wilms tumor in Minnesota. Int J Cancer. 2008 3 15;122(6):1368–73. 10.1002/ijc.23275 18033684

[53] Chu A, Heck JE, Ribeiro KB, Brennan P, Boffetta P, Buffler P, et al Wilms' tumour: a systematic review of risk factors and meta-analysis. Paediatr Perinat Epidemiol. 2010 9;24(5):449–69. 10.1111/j.1365-3016.2010.01133.x 20670226

[54] Schuz J, Schmidt LS, Kogner P, Lahteenmaki PM, Pal N, Stokland T, et al Birth characteristics and Wilms tumors in children in the Nordic countries: a register-based case-control study. Int J Cancer. 2011 5 1;128(9):2166–73. 10.1002/ijc.25541 20607831

[55] Spracklen CN, Wallace RB, Sealy-Jefferson S, Robinson JG, Freudenheim JL, Wellons MF, et al Birth weight and subsequent risk of cancer. Cancer Epidemiol. 2014 10;38(5):538–43. 10.1016/j.canep.2014.07.004 25096278 PMC4188724

[56] Johnson KJ, Carozza SE, Chow EJ, Fox EE, Horel S, McLaughlin CC, et al Parental age and risk of childhood cancer: a pooled analysis. Epidemiology. 2009 7;20(4):475–83. 10.1097/EDE.0b013e3181a5a332 19373093 PMC2738598

[57] Barahmani N, Dorak MT, Forman MR, Sprehe MR, Scheurer ME, Bondy ML, et al Evaluating the Role of Birth Weight and Gestational Age on Acute Lymphoblastic Leukemia Risk Among Those of Hispanic Ethnicity. Pediatr Hematol Oncol. 2015;32(6):382–9. 26237584

[58] Instituto Brasileiro de Geografia e Estatística—IBGE. Estudos e Análises: Informação Demográfica e Socioeconômica Reflexões sobre os Deslocamentos Populacionais no Brasil. Rio de Janeiro, 2011 103p.

